# Moths complement bumblebee pollination of red clover: a case for day-and-night insect surveillance

**DOI:** 10.1098/rsbl.2022.0187

**Published:** 2022-07-13

**Authors:** Jamie Alison, Jake M. Alexander, Nathan Diaz Zeugin, Yoko L. Dupont, Evelin Iseli, Hjalte M. R. Mann, Toke T. Høye

**Affiliations:** ^1^ Department of Ecoscience, Aarhus University, Aarhus, Denmark; ^2^ Arctic Research Centre, Aarhus University, Aarhus, Denmark; ^3^ UK Centre for Ecology and Hydrology, Bangor, UK; ^4^ Institute for Integrative Biology, ETH Zürich, Zürich, Switzerland

**Keywords:** entomology, computer vision, biodiversity, phenology, conservation, Lepidoptera

## Abstract

Recent decades have seen a surge in awareness about insect pollinator declines. Social bees receive the most attention, but most flower-visiting species are lesser known, non-bee insects. Nocturnal flower visitors, e.g. moths, are especially difficult to observe and largely ignored in pollination studies. Clearly, achieving balanced monitoring of all pollinator taxa represents a major scientific challenge. Here, we use time-lapse cameras for season-wide, day-and-night pollinator surveillance of *Trifolium pratense* (L.; red clover) in an alpine grassland. We reveal the first evidence to suggest that moths, mainly *Noctua pronuba* (L.; large yellow underwing), pollinate this important wildflower and forage crop, providing 34% of visits (bumblebees: 61%). This is a remarkable finding; moths have received no recognition throughout a century of *T. pratense* pollinator research. We conclude that despite a non-negligible frequency and duration of nocturnal flower visits, nocturnal pollinators of *T. pratense* have been systematically overlooked. We further show how the relationship between visitation and seed set may only become clear after accounting for moth visits. As such, population trends in moths, as well as bees, could profoundly affect *T. pratense* seed yield. Ultimately, camera surveillance gives fair representation to non-bee pollinators and lays a foundation for automated monitoring of species interactions in future.

## 1. Background

Society's perception of insects is improving, and insect declines are now a primary focus of research in the Anthropocene [1]. Within a growing inventory of ways that insects benefit people, perhaps the most widely recognized is pollination [2]. Insect pollinator declines related to anthropogenic changes threaten not only the yields of important crops [3,4], but also the reproduction of a host of wild plant species [5]. Still, understanding about insect declines remains strongly limited by availability of robust data [6].

Crucially, the data and literature about insect pollinators are imbalanced, and some groups are neglected in the literature [7]. Insect pollination studies are heavily biased towards a small subset of bee species, particularly the social bees in the family Apidae [2]. This is problematic given the major contribution of non-bees to global crop pollination; 38% of crop flower visits were attributed to non-bee insects in a recent meta-analysis [8]. Nocturnal pollinators, e.g. moths, are particularly poorly understood, despite facing additional threats from artificial light at night [9–12]. Evidence is mounting on the agricultural and economic importance of nocturnal pollination, and recent studies showing how apples [13] and avocados [14] benefit from flower visits at night. Still, it remains difficult to generate rigorous, like-for-like comparisons between day- and night-time pollinators [15,16] and too little is known about the scale of nocturnal pollination [17].

*Trifolium pratense* is a plant species that has proven exceptionally useful to study interactions between insect pollinators, wildflowers and crops [3,18,19]. It represents an economically valuable forage legume and provides biological nitrogen fixation for sustainable agriculture [20]. At the same time, *T. pratense* is a functionally important nectar-rich wildflower [21] with particular benefits for wild bumblebees [22]. In Great Britain, the frequency of *T. pratense* has declined by approximately 30% since 1978, offering an explanation for declines in long-tongued bumblebees [22] and shifts in honeybee foraging behaviour [23]. Similarly, shifts in bumblebee community composition since 1940 may have driven declines in *T. pratense* seed yield in Sweden. Although pollen from congeneric flowers has been found on moths on several occasions [11,16,24,25], we find no published study to so much as speculate about moth pollination of *T. pratense*.

Remote cameras show great promise to address data deficiencies in entomology and pollination ecology [26,27]. Here, we demonstrate the use of time-lapse cameras to compare diurnal and nocturnal visitation of *T. pratense* in an alpine grassland. Recording visits with unprecedented precision and continuity, we ask (i) what is the relative visitation rate of bumblebees, moths and other insects to *T. pratense*? (ii) Does consideration of moth visitors, alongside bumblebees, improve predictions of seed set? Finally, to validate whether pollination drives the relationship between visitation and seed set, we ask (iii) does the timing of visits influence the pattern of seed set within each inflorescence? We reveal hitherto undocumented pollination of *T. pratense* by moths—especially *Noctua pronuba*, one of the most abundant macro-moth species in Europe [28]. In doing so, we challenge a century-old doctrine that ‘the only pollinators of red clover … of any consequence are the bees' [29].

## 2. Methods

### (a) Visitation and floral phenology

**Figure 1.**
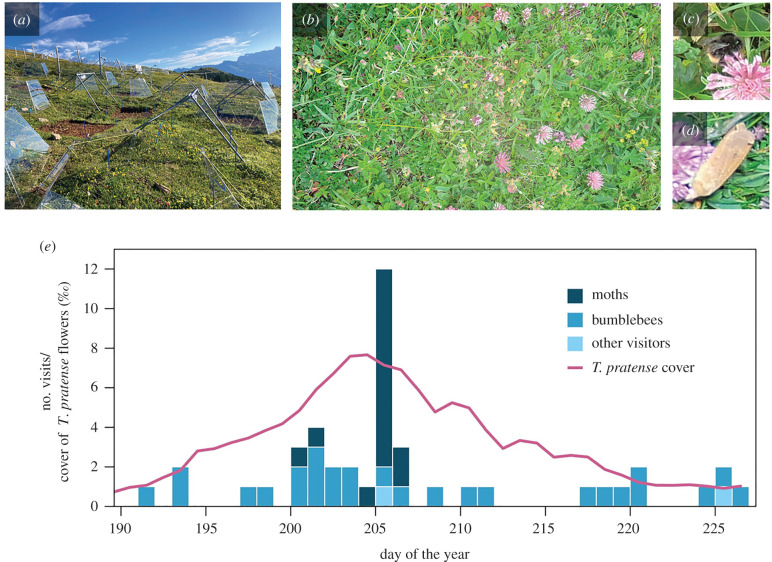
(*a*) Study site in the Calanda region of the Swiss Alps (photo credit: E.I.). (*b*) Image from a time-lapse camera, midday, 20 July 2021. (*c*) Probable *Bombus lapidarius* visit. (*d*) *Noctua pronuba* visit. (*e*) Frequency of *Trifolium pratense* visits from moths (dark blue), bumblebees (blue) and other visitors (light blue), as well as *T. pratense* cover (pink line) recorded by cameras throughout summer 2021.

From 23 June to 15 August 2021, 15 time-lapse cameras (with LED flash) were distributed over approximately 300 m^2^ of semi-natural grassland in an experimental site in the Swiss Alps (figure 1*a,b*). Cameras recorded grassland patches with high representation of flowering forbs, capturing full time series of 36 *T. pratense* inflorescences (mean 2.4 per camera). Nine ‘focused’ cameras recorded at 1 min intervals between 12.00–15.00 and 01.00–03.00, capturing medial periods and reflecting the relative durations of day and night. Six ‘continuous’ cameras recorded at 5 min intervals and were always active. *T. pratense* floral cover was annotated within all midday images from all cameras. Pollinator visits (i.e. images with foraging pollinators) were annotated comprehensively for all 36 *T. pratense* inflorescences that were visible throughout their flowering period. We calculated the floral peak of each *T. pratense* inflorescence as the half-way point between when the last floret emerged and when the first floret senesced. To summarize the timing of visits to each inflorescence relative to peak flowering, we designed a visit lateness index (VLI). The VLI is the mean day of the year (DOY) of visits minus the DOY of the floral peak, and it indicates whether visits occurred before or after peak flowering of an inflorescence. See the electronic supplementary material for further details on image acquisition and annotation.

### (b) Seed set

On 15 August, following dry weather, all *T. pratense* infructescences were collected from under cameras. This was done without prior knowledge of insect visitation. Of 35 collected infructescences, 31 were sufficiently developed to estimate seed set, of which 23 had been successfully recorded on camera throughout the entire flowering period. Before collection, a ‘wand’ (bamboo skewer tipped with a coin-sized card disc) was used to label the infructescence on camera. Infructescences were stored at room temperature in seed envelopes, prior to dissection at Aarhus University in November 2021 to determine seed set. Seed set, denoted *s*, was defined as the number of seeds divided by twice the number of florets, because a floret can produce two seeds [30]. See the electronic supplementary material for the seed head dissection protocol.

During dissection of infructescences, the percentile location of seeded and unseeded florets was recorded. The basal floret location was approximately 0%, while the apical floret location was 100%. To summarize the pattern of seed set across florets in each inflorescence in a parsimonious way, we designed a seed lateness index (SLI). The SLI is the median percentile location of seeded florets (in case of ties, we took the higher of two values), and it indicates whether seeds were more frequent in basal florets (i.e. early-opening florets; low SLI) or apical florets (i.e. late-opening florets; high SLI). For example, a SLI of 30% means that 50% of the seeds were counted in the most basal (i.e. earliest) 30% of florets. SLI is inherently variable for inflorescences with very low seed set (s ≈0). It also converges to 50% for inflorescences with very high seed set (s ≈0.5). As such, inflorescences for which close to half of florets had seeds (s ≈0.25) were weighted more heavily during regression (w=0.25−|s−0.25|).

## 3. Results

### (a) Visitation through time

Across 164 532 images of flowering *Trifolium pratense* recorded at 1 or 5 min intervals, 44 (0.027%) captured foraging pollinators. Of 36 recorded inflorescences, 24 were visited on camera, of which 14 had two or three visits. Moths provided 34% of visits (15) while bumblebees provided 61% (27; figure 1*c–e*). Moth visitation was highly concentrated in time, occurring mostly during the evening and early morning (from 22.00 to 03.00; electronic supplementary material, figure S1) immediately after *T. pratense* cover reached its peak (figure 1*e*). Bumblebees were never present on one inflorescence during consecutive images, while moths were present for over 1 min on three occasions, and over 5 min on one occasion. This indicates that foraging events lasted longer for moths than for bumblebees. Most moths were classified as *Noctua pronuba*, while most bumblebees were classified as a yellow-striped *Bombus* operational taxonomic unit (comprising mostly *B. hortorum*; see the electronic supplementary material for details about visitor identities). There was no positive or negative association between bumblebee and moth visitation (*χ*^2^ = 0.080, d.f. = 1, *p* = 0.78).

### (b) Moth visitation and seed set

**Figure 2.**
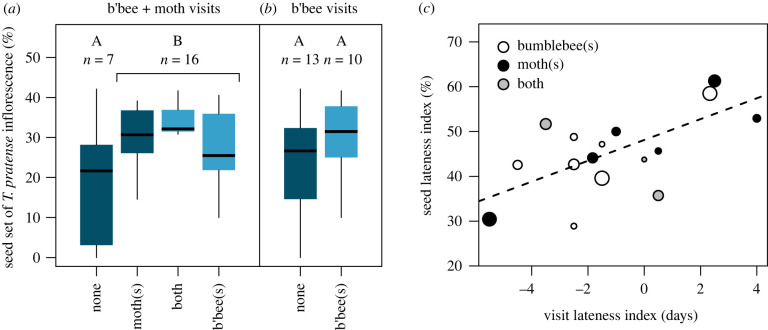
The relationship between visitation and seed set is (*a*) clear when moth visits are accounted for, but (*b*) veiled when only bumblebees are considered. Box colours indicate how visitation categories in (*a*) are merged in (*b*). Letters represent statistical significance within each panel; in (*a*), seed set differs significantly between groups A and B at *p* = 0.040. (*c*) Visit lateness predicts seed lateness in *T. pratense* inflorescences visited by bumblebees (white), moths (black) or both (grey). The VLI represents mean visitation date relative to peak flowering date of an inflorescence. The SLI indicates whether seeds were more frequent in early-opening florets (low SLI) or late-opening florets (high SLI). The dashed line represents a weighted regression (larger points have larger weights), highlighting how inflorescences with early visits had more seeds in early-opening florets (*p =* 0 0.0048).

Seed set ranged from 0 to 42.1% (mean 26.1%) across 31 inflorescences. Of those inflorescences, 23 were visible on camera throughout their flowering period. Inflorescences with bumblebee and/or moth visits on camera had 11.6% higher seed set than those without (linear model: *F*_1,21_ = 4.78, *p* = 0.040, *R*^2^ = 0.186; figure 2*a*). However, if considering only bumblebee visits, no significant difference in seed set was concluded (*F*_1,21_ = 1.39, *p* = 0.252, *R*^2^ = 0.062; ΔAIC = 3.25; figure 2*b*).

### (c) Visit timing and seed set

Inflorescences with a higher VLI had a higher SLI (figure 2*c*; d.f. = 14, *F* = 11.22, *p* = 0.0048, *R*^2^ = 0.405; unweighted regression was still significant with *p* = 0.013). In other words, inflorescences with early visits had more seeds in early-opening florets, implying a causal relationship between visitation and seed set.

## 4. Discussion

Why has moth visitation to *Trifolium pratense* received no mention during a century of dedicated study across Europe and North America [29,30]? We see two principal explanations: (a) studies ignore nocturnal visitation, and (b) moth visitation is negligible. We consider (a) to be true—we can identify no other dedicated *T. pratense* study designed to capture evidence of nocturnal visitation. Even where studies do sample pollinators at night (e.g. [15]), moth visits may be highly concentrated in time (figure 1*e*) and easily missed by traditional methods. We argue (b) is false; our study is unique in its diel and seasonal continuity, and reveals substantial visitation by *Noctua pronuba—*a dominant moth species in Europe [28] that has recently spread across North America [31]. Furthermore, a recent UK study found that moths often carried pollen of the congeneric flower *T. repens*, with more grains on *N. pronuba* than any other moth species [16]. We do not assert that the observed level of moth visitation to *T. pratense* is universal. We do, however, challenge a universal assumption—that bees are the only pollinators of this important wildflower and forage crop species [3,18,20,29,30].

We generate a proof of concept that flower visits on camera can predict the frequency and pattern of seed set (see also [32]), which is a major component of seed yield [30]. Furthermore, *T. pratense* does not set seed without pollen transfer by insects and is one of the most valuable forage legumes worldwide in terms of seed production [20]. Moth visits had an additive impact on seed set (figure 2*a*), so we propose for the first time that moths—alongside bees, genotype, weather and pests [3,18,30]—could affect *T. pratense* seed yield at field scales. Indeed, we offer an explanation as to why a previous pollination study in the same region of the Swiss alps found no relationship between daytime visitation and seed set in *T. pratense* [33]. We cannot completely rule out effects of e.g. seed predation by weevils in the present study [18]. However, continuous surveillance allowed us to relate the timing of pollinator visits to the pattern of seed set within each inflorescence. Specifically, we found that inflorescences with early visits had more seeds in early-opening florets (figure 2*c*). This strongly suggests that pollination drives the observed relationship between visitation and seed set, rather than any confounding variable.

Clearly camera surveillance can provide a temporally representative view of flower visitation [27], which is difficult to achieve through visual observations [15]. Pollen microscopy and DNA metabarcoding reveal the types of pollen carried by nocturnal insects [14,16,25], while exclusion experiments establish which agricultural plants depend on nocturnal pollination [13]. However, the scale of nocturnal pollination has proven particularly difficult to quantify [17], and cameras can provide robust estimates of relative visitation by day- and night-active pollinators [32]. This highlights not only which pollinators contribute to plant reproduction, but which floral resources sustain insect populations. *T. pratense* is already highly recommended by experts for bumblebee-friendly agri-environmental seed mixtures [22], while late-season mass-flowering *T. pratense* can be crucial for bumblebee reproduction [34]. We reveal that nectar provision by *T. pratense* may also benefit nocturnal Lepidoptera. Ultimately, cameras should play a central role in future monitoring schemes for plant–pollinator interactions. Standardized image libraries can provide a permanent archive of plant and insect phenology, and train deep-learning models to automatically extract ecological information [26,35,36]. Using both cameras and established methods, science will converge on the true extent of pollinator declines—but also the most appropriate remedial interventions.

## Data Availability

The data and code that support the findings of this study are available in the electronic supplementary material [37].
